# Incidence of Infection in *Prnp* ARR/ARR Sheep following Experimental Inoculation with or Natural Exposure to Classical Scrapie

**DOI:** 10.1371/journal.pone.0091026

**Published:** 2014-03-10

**Authors:** Martin Jeffrey, Stuart Martin, Francesca Chianini, Samantha Eaton, Mark P. Dagleish, Lorenzo González

**Affiliations:** 1 Animal Health and Veterinary Laboratories Agency (AHVLA-Lasswade), Pentlands Science Park, Bush Loan, Penicuik, Midlothian, Scotland, United Kingdom; 2 Moredun Research Institute, Pentlands Science Park, Bush Loan, Penicuik, Midlothian, Scotland, United Kingdom; University of Edinburgh, United Kingdom

## Abstract

The prion protein gene (*Prnp*) is highly influential in determining risk and susceptibility of sheep exposed to classical scrapie. Sheep homozygous for alanine at codon 136 and arginine at codons 154 and 171 (ARR/ARR) of the *Prnp* gene are historically considered to be highly resistant to classical scrapie, although they form a significant fraction of cases of atypical scrapie. To date, experimental transmission of prions to ARR/ARR sheep has only been achieved with the BSE agent and mostly by the intracerebral route. We summarise here the results of six separate studies, in which 95 sheep of the ARR/ARR genotype were naturally exposed to (n = 18) or experimentally challenged with (n = 77) natural or experimental sources of classical scrapie by the oral, intra-intestinal, subcutaneous or intracerebral routes and allowed to survive for periods of up to 94 months post-infection. Only the intracerebral route resulted in disease and/or amplification of disease associated PrP (PrP^d^), and only in two of 19 sheep that survived for longer than 36 months. Discriminatory immunohistochemistry and Western blot confirmed the scrapie, non-BSE signature of PrP^d^ in those two sheep. However, the neuropathological phenotype was different from any other scrapie (classical or atypical) or BSE source previously reported in sheep of any *Prnp* genotype. These studies confirm the widely held view that ARR/ARR sheep are highly resistant to classical scrapie infection, at least within their commercial lifespan. Moreover, within the constraints of the present studies (only two infected sheep), these results do not support the suggestion that atypical scrapie or BSE are generated by adaptation or mutation of classical scrapie in sheep of resistant ARR/ARR genotype.

## Introduction

The transmissible spongiform encephalopathies (TSEs) are characterised by the accumulation of abnormal forms of a host-coded, cell membrane sialoglycoprotein called prion protein (PrP). Scrapie, or classical scrapie, of sheep and goats is the archetypal TSE and has been recognised as contagious outbreaks of disease for several centuries. More recently, a novel, apparently non-contagious or sporadic form of sheep TSE, originally called Nor 98 [1] and now more commonly referred to as atypical scrapie, has been recognised in Europe and elsewhere.

The prion protein gene (*Prnp*) controls susceptibility to both atypical and classical scrapie [2]. Sheep bearing alanine (A) or valine (V) at codon 136 and glutamine (Q) at codon 171 of PrP are susceptible to classical scrapie. In contrast, classical scrapie is rarely reported in sheep homo- or hetero-zygous for the allele that bears A at codon 136 and arginine (R) at codons 154 and 171. In separate UK studies, sheep scrapie was not identified in aged ARR/ARR or ARQ/ARR sheep in flocks or geographical regions with endemic scrapie [3], [4], [5], [6]. Other epidemiological studies also show that scrapie is very rare in ARQ/ARR sheep and absent from ARR/ARR sheep in the UK [7]. However, single cases of natural scrapie infection in ARR/ARR sheep have been reported from Germany, France [8] and possibly also Japan [9].

In contrast, atypical scrapie is relatively common in sheep that are homozygous or heterozygous for ARR alleles but is rare in genotypes considered highly susceptible to classical scrapie such as the VRQ/VRQ genotype [10]. In addition, ARR/ARR sheep succumb to cattle and sheep BSE infection following intracerebral challenge, albeit with extended incubation periods relative to homozygous ARQ sheep [11], [12]. ARR/ARR sheep orally [13] or intra-splenically [14] dosed with BSE may also sustain infection though development of clinical disease was not achieved by these routes.

Over the last two decades, we have performed several experiments in which ARR/ARR sheep have been exposed to natural scrapie or have been experimentally challenged by different routes. The purpose of this report is to draw together the data from those studies and report the susceptibility of ARR/ARR sheep to classical scrapie.

## Materials and Methods

All studies, including experimental inoculations, care of animals and euthanasia, were carried out in accordance with the UK Animal (Scientific Procedures) Act 1986. Studies 1 and 3–6 were performed at the Moredun Research Institute under licenses from the UK Government Home Office number 60/2656 (renewed in 2005 with number 60/3646). Study 2 was carried out at the Agricultural Development and Advisory Service facilities at High Mowthorpe under project license number 70/5155. Animals were monitored daily for the presence of neurological signs compatible with scrapie and were euthanized once those signs reached a standard, pre-determined end point (for details refer to [15]), when showing signs of intercurrent disease unresponsive to treatment, or for welfare reasons. In most cases, however, sheep were killed at the scheduled termination of the different studies. In all cases, euthanasia was performed by intravenous injection of barbiturate overdose followed by exsanguination.

### ARR/ARR sheep included in the different studies

**Figure 1:**
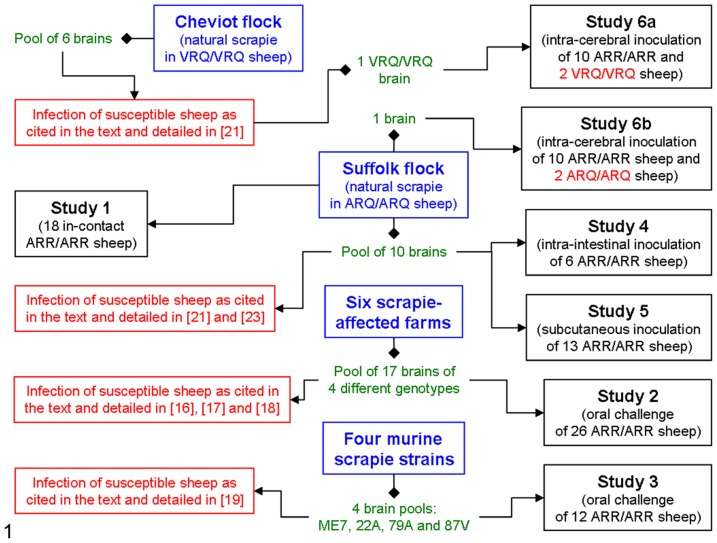
Design of the different studies with reference to the source of the inocula used. In green, inocula used; in blue, source of the inocula; in black use of the inocula for the six different studies on ARR/ARR sheep; in red, use of the same inocula to challenge sheep of susceptible genotypes.

Ninety-five ARR/ARR sheep were included in the following six studies (Table 1 and Fig. 1):

**Table 1:** Design of the different studies.

Study	Exposure	Source	Dose	Months post-infection or age at post-mortem examination								Total
No.				0–12	13–24	25–36	37–48	49–60	61–72	73–84	85–96	
**1**	Natural	ARQ/ARQ	n/a					9	5	1	3	18
**2**	Oral	Sheep pool	5		3	3	3	3	3		11	26
**3**	Oral	Murine strains	5			6	6					12
**4**	Intraintestinal	ARQ/ARQ	1		1		1		4			6
**5**	Subcutaneous	ARQ/ARQ	0.1 (+2*)		7	2					4*	13
**6a**	Intracerebral	ARQ/ARQ	0.1				2		4	4		10
**6b**	Intracerebral	VRQ/VRQ	0.1	1					1	8		10
**Total**				1	11	11	12	12	17	13	18	95

Age in months for naturally exposed sheep of study 1; months post-infection for all other studies. Sheep pool, pool of 17 scrapie brains (VRQ/VRQ = 6, VRQ/ARQ = 6, ARQ/ARQ = 4, VRQ/ARR = 1).

Murine strains, sheep dosed with 22 A or 87 V killed at 35 mpi; sheep dosed with ME7 or 79 A killed at 47 mpi. Dose in grams: n/a, not applicable;

*, four sheep were boosted with 2 g of inoculum by the subcutaneous route (months post-inoculation correspond to those of original challenge).

#### Study 1 (Natural infection)

In a closed flock of Suffolk sheep, in which the average incidence of scrapie in ARQ/ARQ sheep over a nine-year period was 84% [15], 18 ARR/ARR sheep were allowed to survive for over 48 months (Table 1 and Fig. 1). These ARR/ARR sheep were reared from birth in continuous contact with scrapie infected individuals.

#### Study 2 (Oral infection with sheep scrapie)

26 ARR/ARR Cheviot lambs were orally dosed at 2–3 weeks of age on five consecutive days with 1 g of a brain pool homogenate (total dose 5 g) and were allowed to survive for 14 to 92 months post-infection (mpi; Table 1 and Fig. 1). The brain pool homogenate (RBP1) was made from whole brains taken from 17 scrapie affected sheep of five different breeds and four *Prnp* genotypes (VRQ/VRQ, VRQ/ARQ, ARQ/ARQ and VRQ/ARR), which originated from six different farms. After challenge with the same 5 g oral dose, this inoculum produced attack rates of 100% in VRQ homozygous sheep [16], [17] and of 64% in ARQ/ARR sheep [18]. A 1 g dose of the same inoculum administered also by the oral route gave rise to 100% attack rates in VRQ/VRQ, VRQ/ARQ, VRQ/ARR and ARQ/ARQ sheep [17], [18].

#### Study 3 (Oral infection with experimental murine-adapted scrapie)

A total of 12 Suffolk or Cheviot ARR/ARR sheep between the ages of 3–5 months were orally dosed with 25 ml of a 20% suspension of brain from scrapie affected mice (5 g tissue equivalent). Three sheep each were infected with the murine adapted strains ME7, 22 A, 79 A, or 87 V, all of which were originally derived from scrapie affected sheep, and killed between 35–47 mpi (Table 1 and Fig. 1). The rationale for this experiment and the results of challenging sheep of other genotypes with the same murine strains were reported by Sisó *et al*., [19]. Briefly, oral challenge of VRQ/VRQ sheep with the same 5 g dose induced 100% attack rate with ME7, 50% with 22 A, 33% with 79 A and 0% with 87 V. Attacks rates for the same dose and route in ARQ/ARQ sheep (some of which were polymorphic at codons 112, 141, or 168) were 86% with ME7, 83% with 22 A, 17% with 79 A and 0% with 87 V. All four murine scrapie strains produced 100% attack rates in VRQ/VRQ and ARQ/ARQ sheep when administered by a combined oral, subcutaneous and intracerebral route.

#### Study 4 (Inoculation of intestinal loops with sheep scrapie)

Single isolated gut loops were created as described previously [20] in six two month-old, scrapie-free ARR/ARR Suffolk lambs. Loops were created to include the distal ileum with its continuous Peyer's patch and inoculated with 5 ml of a Suffolk scrapie brain 20% homogenate (1 g tissue equivalent). Two sheep were killed at 16 and 47 mpi because of intercurrent health problems; the remaining 4 sheep were healthy when killed at the end of the experiment at 70–72 mpi (Table 1). The inoculum was sourced from scrapie confirmed clinical cases (pool of 10 ARQ/ARQ sheep) from the naturally infected Suffolk flock described above (study 1, see Fig. 1). In a different experiment, the same inoculum was used to infect sheep of the VRQ/VRQ, VRQ/ARQ and ARQ/ARQ genotypes either by the oral or subcutaneous routes with 100% attack rates in all cases [21].

#### Study 5 (Sub-cutaneous inoculation with sheep scrapie)

13 New Zealand-derived ARR/ARR Suffolk lambs were subcutaneously inoculated at 6 months of age with 1 ml of a 10% clarified homogenate of the same inoculum as used in study 4 (0.1 g tissue equivalent; Fig.1). The injection was done in the drainage area of the right prefemoral lymph node, as described in detail previously [22]. Seven ARR/ARR sheep were killed at 22 mpi and two at 29 and 32 mpi; the remaining four were re-challenged subcutaneously at 32 mpi with 10 ml (5 ml in each flank) of a 20% dilution of the same inoculum (2 g tissue equivalent) and killed 58 to 62 months after the second inoculation (90 to 94 months after the original challenge; Table 1). The inoculum used induced infection in 100% ARQ/ARQ Suffolk sheep without threonine polymorphism at codon 112 when injected by the same subcutaneous route [23].

#### Study 6 (Intracerebral inoculation with sheep scrapie)

In study 6a, 10 ARR/ARR Suffolk sheep were intracerebrally challenged at four months of age with 0.5 ml of a 20% sheep scrapie brain homogenate (0.1 g tissue equivalent) using a scrapie brain homogenate derived from an ARQ/ARQ sheep from the naturally infected Suffolk flock described above (Fig. 1). Two sheep were found dead at 39 and 43 mpi, another four were culled at 61 to 72 mpi due to welfare issues and the remaining four were killed at the end of the experiment (79 mpi; Table 1). Two age-matched ARQ/ARQ sheep were also inoculated (same inoculum, route and dose) and died at ∼17 mpi with a pathological and biochemical scrapie phenotype indistinguishable from that of the inoculum donor and other ARQ/ARQ sheep in their flock.

In study 6b, 10 ARR/ARR Cheviot sheep were also intracerebrally challenged at the same age and with the same dose as the Suffolk sheep but with an inoculum derived from a VRQ/VRQ Cheviot sheep that succumbed to confirmed scrapie after oral infection with a brain pool homogenate of six natural scrapie cases in sheep of the same breed and genotype all derived from the same flock (Fig. 1; for details of this source see [21]). Two sheep were found dead at 11 and 72 mpi, one was killed with terminal neurological signs at 74 mpi and the remaining seven were culled at the end of the experiment (79 mpi; Table 1). Two age-matched VRQ/VRQ sheep were also inoculated (same inoculum, route and dose) and died at ∼5 mpi with a pathological and biochemical scrapie phenotype that was indistinguishable from that of the donor sheep.

### Laboratory examinations

From each of the above studies, a detailed necropsy was performed and samples of lymphoid tissues, digestive tract, brain and spinal cord, peripheral and autonomic nervous system tissues, striated muscles and other organs were taken for immunohistochemical (IHC) examinations, as detailed in table 2. Tissues were processed to paraffin wax and stained with haematoxylin and eosin (brain only) or subjected to IHC labelling for disease associated PrP (PrP^d^) using R145 monoclonal antibody (binding to ovine PrP amino acid [aa] sequence 222–226 [24]) as described previously [11]. Two additional PrP antibodies, F99 (aa sequence 220–225 [25]) and 3F10 (aa sequence 137–151 [26]) were used in serial sections to help confirming low levels of PrP^d^ accumulation found with R145. In addition, 2A11 (aa sequence 163–171 [27], and SAF 84 (aa sequence 166–172 [28]), monoclonal antibodies that do not recognize R at codon 171 were also used on tissue sections where positive PrP^d^ labelling was detected with R145.

**Table 2:** Details of tissues examined routinely by IHC in ARR/ARR sheep of the different studies.

Tissue	Study number					
	1	2	3	4	5	6
**Lymphoid tissues**						
Pharyngeal tonsil		X				
Palatine tonsil	X	X	X	X	X	X
Nictitating membrane		X			X	X
Medial retropharyngeal LN	X	X	X	X		X
Lateral retropharyngeal LN		X				
Submandibular LN		X			X	X
Parotid LN		X				
Prescapular LN		X			X	X
Tracheobronchial LN		X				
Mediastinal LN		X				
Mesenteric LN		X	X		X	X
Ileocecal LN		X				
Inguinal LN		X			X	
Popliteal LN		X			X	
Spleen		X	X		X	X
**Digestive tract**						
Oesophagus		X				
Rumen		X				
Reticulum		X				
Omasum		X				
Abomasum		X				
Duodenum		X				
Jejunum		X		X	X	
Ileum	X	X	X		X	X
Caecum		X				
Colon		X			X	
Rectum	X	X			X	X
**Central nervous system**			X	X	X	X
Frontal cortex			X	X	X	X
Corpus striatum	X		X	X	X	X
Thalamus/hypothalamus				X		
Hippocampus			X	X	X	X
Midbrain			X	X	X	X
Cerebellum		X	X	X	X	X
Medulla oblongata			X	X	X	X
Obex	X	X	X	X	X	X
Cervical spinal cord			X		X	X
Thoracic spinal cord			X		X	X
Lumbar spinal cord			X		X	X
Retina					X	X
**Peripheral nervous system**						
Trigeminal G		X			X	X
Nodose G		X			X	X
Cranial cervical G		X				
Stellate G		X				X
Sympathetic chain		X			X	X
Vago-sympathetic trunk		X				
Vagus nerve		X			X	X
Cranial mesenteric G		X			X	X
Radial nerve		X				
Sciatic nerve					X	X
**Muscles and other organs**						
Semitendinous muscle					X	X
Intercostal muscle					X	X
Occular muscles					X	X
Tongue						X
Heart	X	X			X	
Lung		X				
Liver		X				
Kidney		X				
Adrenal gland		X			X	X

LN, lymph node; G, ganglion.

Most digestive tract tissues provided opportunity to examine both nervous and lymphoid components.

Tissues examined in each study (for description of each study refer to text) are marked with an X.

Western blotting (WB) was carried out in samples of medulla oblongata and/or cerebellum of all sheep using P4 (aa sequence 93–99 [29]) and SAF84 PrP antibodies as described previously [21] to detect protease resistant PrP (PrP^res^). In addition, samples of five different brain areas from the only sheep that developed clinical scrapie were examined with L42 (aa sequence 148–153 [28]) and F99 PrP monoclonal antibodies.

The Bio- Rad TeSeE ELISA is the screening test used in the current statutory UK small ruminant TSE surveillance programme, and was used to test cerebellum samples from all 20 intracerebrally challenged ARR/ARR sheep and the four controls of that experiment (study 6).

## Results

### Attack rates

None of the 64 sheep surviving for less than 72 months after scrapie challenge showed any post-mortem indication of PrP^d^ accumulation. These included 14 naturally exposed sheep, 27 orally dosed (including the 12 dosed with murine scrapie), all six receiving intra-intestinal inoculation, nine injected subcutaneously and six infected intracerebrally. Among the remaining 33 sheep, which were aged or survived for 72 or more mpi, none of the four naturally exposed, the 11 orally dosed with sheep scrapie or the four inoculated subcutaneously were positive for PrP^d^/PrP^res^, either by IHC or WB, in any of the tissues examined. Of the 14 sheep challenged by the intracerebral route only two, one Cheviot and one Suffolk sheep, showed PrP^d^ accumulation, as described below. In summary, these results indicate attack rates of 0% in sheep naturally exposed or experimentally infected by routes other than the intracerebral, regardless of their survival time. For intracerebrally challenged sheep, attack rates would vary between 10% (2/20), if all inoculated sheep are considered, and 14.3% (2/14), if only those coeval or older than the first indication of infection (72 mpi) are accounted for.

### PrP^d^ positive ARR/ARR Suffolk sheep

**Figure 2:**
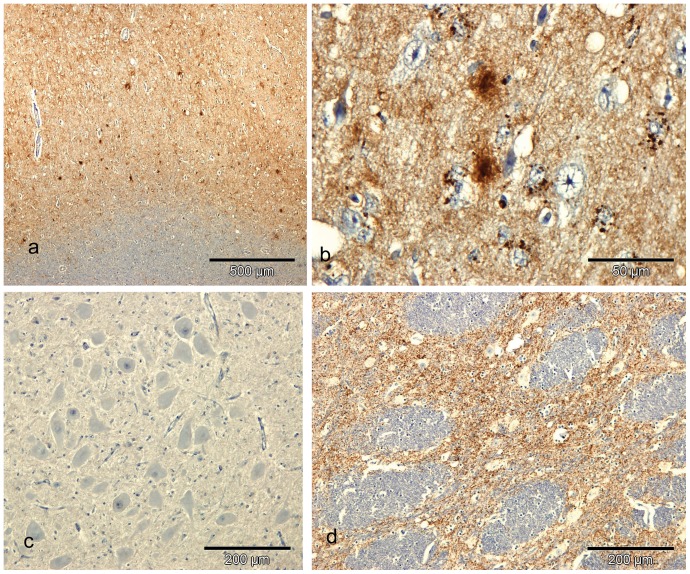
PrP^d^ accumulation in CNS tissues of clinically affected and pre-clinical ARR/ARR sheep. a) Cerebral cortex of an ARR/ARR Cheviot sheep challenged with a VRQ/VRQ scrapie source showing marked diffuse particulate PrP^d^ accumulation in grey matter with multifocal mini–plaque like accumulations. Bar  = 500 µm. IHC with R145 PrP antibody and haematoxylin counterstaining. b) Detail of the cerebral cortex of the ARR/ARR scrapie affected Cheviot sheep showing diffuse, marked intra-astrocytic PrPd and multifocal intense mini-plaque–like PrP^d^ accumulations. Bar  = 50 µm. IHC with R145 PrP antibody and haematoxylin counterstaining. c) Pre-clinical ARR/ARR Suffolk sheep challenged with ARQ/ARQ scrapie sources. Note absence of detectable PrP^d^ accumulation in the dorsal motor nucleus of the vagal nerve. Bar  = 200 µm. IHC with R145 PrP antibody and haematoxylin counterstaining. d) Pre-clinical ARR/ARR Suffolk sheep showing diffuse grey matter PrP^d^ accumulation in the thalamus. Bar 200 µm. IHC with R145 PrP antibody and haematoxylin counterstaining.

One neurologically unremarkable ARR/ARR Suffolk sheep intracerebrally challenged with ARQ/ARQ Suffolk scrapie was culled at 72 mpi because of persistent lameness and problems related to its hooves. On histological examination of the brain this sheep showed neuropil vacuolation in the thalamus. Immunohistochemistry confirmed PrP^d^ accumulation in the thalamus (Fig. 2a), the midbrain, the parietal cerebral cortex and the obex. PrP^d^ was almost exclusively present in the form of particulate accumulation within the neuropil. In the obex, sparse particulate deposits of PrP^d^ were present in the spinal tract of the trigeminal nerve but not in the dorsal motor nucleus of the vagus nerve (Fig. 2b). Immunoreactivity to R145 antibody was confirmed by positive immunolabelling with F99 and 3F10 but no PrP^d^ was detected with either SAF 84 or 2A11, confirming the ARR variant of the protein.

PrP^res^ was not detected by WB or ELISA done on brain samples and all other tissues examined by IHC were also negative.

### PrP^d^ positive ARR/ARR Cheviot sheep

**Figure 3:**
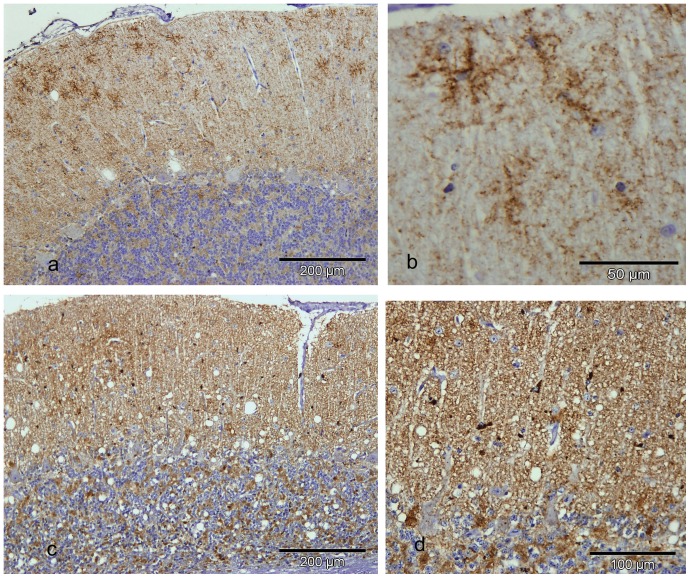
PrP^d^ labelling and vacuolation in the cerebellar cortex of VRQ/VRQ and ARR/ARR sheep. a) VRQ/VRQ sheep showing diffuse particulate and stellate types of PrP^d^ accumulation in the cerebellar molecular layer. Vacuolation is relatively subtle. Bar 200 µm. IHC with R145 PrP antibody and haematoxylin counterstaining. b) VRQ/VRQ sheep showing detail of the stellate type of PrP^d^ accumulation centred on glial cell nuclei in the molecular layer of the cerebellum. Bar 50 µm. IHC with R145 PrP antibody and haematoxylin counterstaining. c) ARR/ARR sheep showing diffuse particulate but not stellate PrP^d^ accumulation in both molecular and internal granule cell layers and prominent intra-glial types of PrP^d^ accumulation in the cerebellar molecular layer. Vacuolation is abundant. Bar 200 µm. IHC with R145 PrP antibody and haematoxylin counterstaining. d) ARR/ARR sheep showing detail of the intensity of particulate type of PrP^d^ accumulation and intense granular PrP^d^ accumulation associated with glial cell nuclei (intra-microglial type) in the cerebellar molecular layer. Bar 100 µm. IHC with R145 PrP antibody and haematoxylin counterstaining.

One ARR/ARR Cheviot sheep intracerebrally challenged with VRQ/VRQ Cheviot scrapie collapsed after a short clinical course of 3 weeks, in which the animal displayed vague neurological signs, and was killed at 75 mpi. The brain showed severe vacuolation throughout all grey matter regions and PrP^d^ was also present throughout all neuroanatomical areas of the brain and spinal cord. A wide range of PrP^d^ types [30] were present including types consistent with intra-neuronal and intra-glial and several extracellular types. Most of the latter were in the form of coarse or fine diffuse particulate PrP^d^ in the grey matter neuropil, while glial associated perivascular aggregates were infrequent. In the thalamus and cerebral cortex, distinctive, multifocal, intensely-labelled mini plaque-like deposits and marked intra-astrocytic PrP^d^ accumulations were observed (Fig. 2c,d). Labelling was absent when R145 positive areas were incubated with antibodies 2A11 or SAF 84. Neither the pattern of vacuolation nor the pattern and distribution of PrP^d^ accumulation was consistent with that of the VRQ/VRQ Cheviot donor or the two positive control sheep. These showed less intense and less widespread vacuolation (Fig. 3a,b), predominant stellate type of PrP^d^ and absence of mini plaques, although the intra-glial, intra-neuronal and diffuse particulate PrP^d^ types were in common with ARR/ARR sheep (Fig. 3c,d).

**Figure 4:**
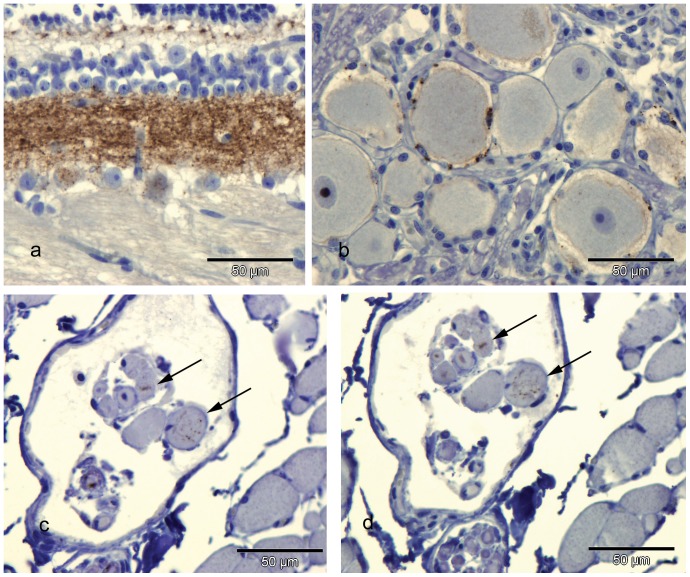
Additional tissues of ARR/ARR clinically affected Cheviot sheep showing PrP^d^ accumulation. a) Retina showing diffuse PrP^d^ accumulation in outer plexiform layer and granular accumulations in inner plexiform layer and retinal ganglion cells. Bar = 50 µm. IHC with R145 PrP antibody and haematoxylin counterstaining. b) Trigeminal ganglion showing granular PrP^d^ accumulation in satellite cells Bar = 50 µm. IHC with R145 PrP antibody and haematoxylin counterstaining. c) Muscle spindle from ocular muscle showing weak granular PrP^d^ accumulation in intrafusal muscle fibres (arrows). Bar = 50 µm. IHC with R145 PrP antibody and haematoxylin counterstaining. d) The same muscle spindle as in c) labelled with anti- PrP antibody F99. The same intrafusal muscle fibres as with R145 are labelled (arrows). Bar = 50 µm. IHC with F99 PrP antibody and haematoxylin counterstaining.

PrP^d^ accumulation was not detected in the lymphoid system, digestive tract or in most of the peripheral nervous system and other tissues examined. In the retina, diffuse PrP^d^ labelling was found in the outer plexiform layer and coarse particulate deposits in the inner plexiform layer and in the soma of retinal ganglion cells (Fig 4a). Low levels of PrP^d^ accumulation were found in the trigeminal ganglion's satellite cells (Fig 4b) and in intrafusal fibres of the infra-orbital muscles (Fig 4c). In trigeminal ganglion peri-axonal PrP^d^ accumulation was detected in myelinated axons. Incubation of serial sections with F99 (Fig 4d) and 3F10 confirmed the specificity of labelling in these tissues.

**Figure 5:**
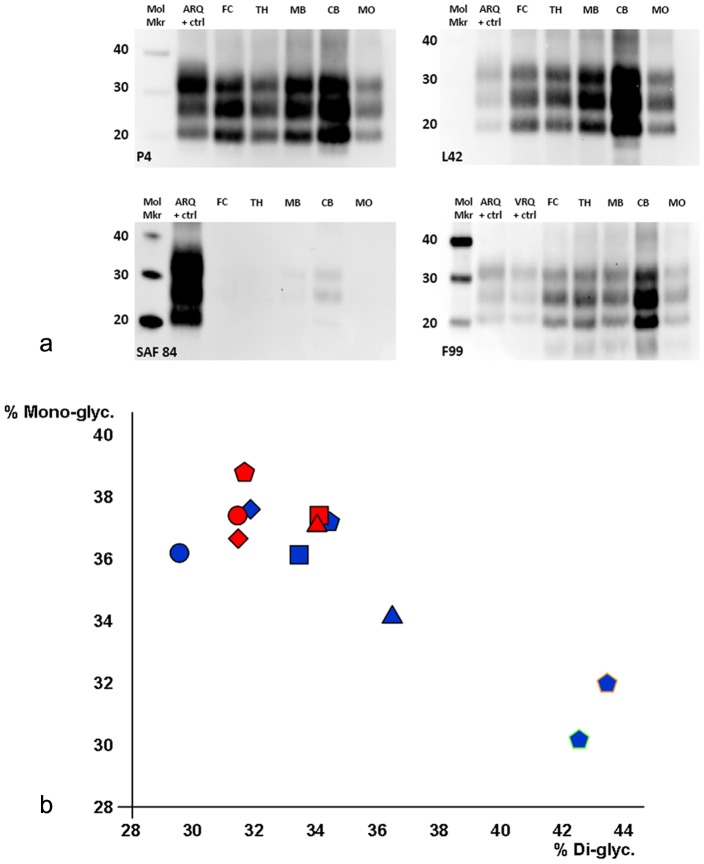
Western blotting of different brain areas of ARR/ARR clinically affected Cheviot sheep. a) Western blots of brain tissue with 4 different PrP antibodiesImmunoblots of frontal cortex (FC) thalamus (TH) midbrain (MB) cerebellum (CB) and medulla at the level of the obex (MO) each showed significant accumulation of PrP^res^ with antibodies P4, L42, and F99. With each of these antibodies the cerebellum showed the greatest concentration of PrP^res^. Compared with ARQ/ARQ and VRQ/VRQ scrapie positive control obex samples, the mono-glycosylated band predominates in most of the ARR/ARR samples. The unglycosylated band from all ARR/ARR and positive control samples has a molecular weight of ∼20 to 21 kDa. No labelling or only trace labelling was found when the antibody SAF84 was used. Mol mkr: molecular weight marker (Note: the molecular weight marker produced a very faint signal with L42. To position the weight reference values the blot was digitally overexposed but it is the original, non-saturated blot that is reproduced). b) Graph showing the proportion of di-glycosylated and mono-glycosylated PrP^res^ for brain from the clinically affected ARR/ARR sheep in comparison with ARQ/ARQ and VRQ/VRQ controls. For each of the brain sites the mono-glycosylated fraction of PrP^res^ was present in a relatively greater amount than the di-glycosylated fraction when labelled with either P4 or L42 antibodies. In contrast both VRQ/VRQ control and ARQ/ARQ positive controls had a relatively greater amount of di-glycosylated PrP^res^. Values for P4 antibody are shown in blue and for L42 in red; frontal cortex, diamonds; thalamus, squares; midbrain, triangles; cerebellum, circles; obex, pentagons; the Suffolk sheep ARQ/ARQ positive control (obex) is outlined in orange and the Cheviot sheep VRQ/VRQ positive control (obex) is outlined in green.

Western blotting of brain samples incubated with P4, L42 or F99 (Fig. 5a) confirmed the presence of PrP^res^ in the obex, frontal cortex, thalamus, midbrain and cerebellum. The highest signal was detected in cerebellum. In almost all brain areas tested the strongest PrP^res^ signal was obtained from the monoglycosylated fraction, which contrasted with the strongest signal of the diglycosylated fraction in the VRQ/VRQ donor and control recipients (Figs. 5a and 5b). The mobility of the unglycosylated fraction corresponded to a molecular weight of ∼20 to 21 kDa, similar to that of the VRQ/VRQ and ARQ/ARQ controls and consistent with classical scrapie. When the same brain samples were incubated with SAF84 (Fig. 5a), no signal or only trace signals were obtained for any of the bands. These observations are in agreement with the 2A11 and SAF84 IHC results, and indicate that PrP^res^ was of the ARR variant.

The Bio-Rad TeSeE ELISA gave positive test values in brain samples from VRQ/VRQ and ARQ/ARQ positive scrapie controls and from the clinically affected ARR/ARR Cheviot sheep.

## Discussion

In agreement with previously published studies, the above experiments show that ARR/ARR sheep are extremely resistant to classical scrapie when naturally exposed to a highly contaminated environment or when infected by experimental protocols that approximate natural exposure. However, experimental intra-cerebral challenge shows that it is possible to induce disease in sheep of this genotype, albeit with low attack rates and incubation periods that are years longer than those in sheep of susceptible genotypes. Therefore, these data provide proof of principle that ARR/ARR sheep are susceptible to UK classical sheep scrapie sources albeit only by a highly artificial and efficient route of challenge.

The experimental gut loop protocol circumvents transit of orally dosed infectivity through the upper part of the alimentary system, avoiding enzymatic degradation of PrP^d^. It thus maximises the opportunity of transport of infectivity across the intestinal barrier to permit amplification of infectivity in gut associated lymphoid tissues of susceptible sheep [20]. Despite this and a relatively large dose, this route did not result in generalised or even localised amplification of PrP^d^ in ARR/ARR sheep. Equally, oral and subcutaneous infection did not result in transmission of infection. Failure to induce classical scrapie using these routes of inoculation is more relevant to natural disease than the intra-cerebral route, particularly when considering that the volumes of inocula used were in excess of those likely to be found under environmental conditions of even the highest infectivity pressure. The lack of transmission in these experiments is consistent with the absence of infection in ARR/ARR sheep exposed for prolonged periods to a highly contaminated environment (the “natural infection” group) and with many other epidemiological studies of natural scrapie in the UK [3], [4], [5], [6], [7] and elsewhere [31] which do not report classical scrapie in ARR/ARR sheep.

However, single cases of classical scrapie have been reported in ARR/ARR sheep from Japan [9], Germany and France [8], and in all instances by the detection of PrP^res^ in Western blot analyses of brain samples. The Japanese case, a Suffolk sheep, appeared to be clinically affected but no data are available about the clinical or pathological phenotype of the disease. The German case, a black-headed German mutton sheep, was apparently healthy and the French case, a 5 year-old sheep, showed neurological signs of histopathologically confirmed listeriosis. Detailed data on clinico-pathological phenotypes are not available for either case, although the French case was successfully transmitted to Tg338 mice [8]. These reports suggest that cases of classical scrapie may occur naturally in ARR/ARR sheep, although all epidemiological and experimental evidence indicate that such occurrences would be sporadic or exceptional. However, more frequent detection of such cases may be hampered by current sampling strategies which focus on the hind brain, as shown by the failure of statutory ELISA testing to detect the single pre-clinically affected intra-cerebrally challenged Suffolk sheep in the present study.

ARR/ARR sheep also appear to be much more resistant to infection with classical scrapie than with experimental sheep BSE. Thus, ARR/ARR sheep intra-cerebrally challenged with 0.5 mg tissue equivalent of sheep BSE (i.e., allowing for potential differences in infectious titre, a 500 times smaller dose than in the scrapie experiment reported here) developed clinical disease with a 100% attack rate and a survival time of 49±5 months [12]. Similarly, ARR/ARR sheep intra-cerebrally challenged with cattle BSE showed figures of 56% attack rate and 49±13 months survival time (Houston et al., unpublished observations). Moreover, susceptibility of ARR/ARR sheep to sheep BSE by the oral route [13] and to cattle BSE by the intra-splenic route [14] has been documented, while oral, intra-intestinal and sub-cutaneous challenge with sheep scrapie and murine adapted sheep scrapie failed to transmit infection, at least as judged by PrP^d^ or PrP^res^ detection. The difference in pathogenicity of BSE and scrapie agents for ARR/ARR sheep does not appear to be a property of the agents *per se* as this difference in pathogenicity is not observed in sheep of other genotypes. For example, oral infection of ARQ/ARQ sheep (without polymorphisms at codons 112 or 141) with ARQ/ARQ sheep scrapie results in complete attack rates and short survival times of 23±2 months [21], while sheep of the same genotype inoculated by the same route and with the same 5 g dose of ARQ/ARQ sheep BSE also showed a 100% attack rate and survival times of 23±2 months (first passage) or 25±2 months (second passage) [32]. Similarly, oral infection of VRQ/VRQ sheep with ARQ/ARQ sheep scrapie produces a 100% attack rate with survival times of 47±7 months [21] and infection of sheep of the same genotype with cattle BSE by the same route and dose also results in complete attack rate figures and survival times of ∼59 months (Jeffrey et al., unpublished observations). It is worth pointing out that despite the studies reported here using a variety of natural and experimental scrapie sources, all of which proved to be infectious for sheep of susceptible *Prnp* genotypes [15], [16], [17], [18], [19], [21], [23], the number of such sources was limited. We cannot therefore rule out the possibility that other sources or strains of classical scrapie could result in higher attack rates or different disease phenotypes in ARR/ARR sheep, as is found with the BSE agent.

Although the pathological and biochemical features of the single clinical scrapie case in an ARR/ARR genotype in our studies was consistent with classical rather than atypical scrapie or BSE, the disease phenotype was dissimilar to the VRQ/VRQ case that provided the inoculum and to the two VRQ/VRQ positive control sheep challenged with the same inoculum. The pathological phenotype of this single ARR/ARR scrapie case has little resemblance to any other classical scrapie source the authors have examined in any sheep breed or *Prnp* genotype. This suggests that although some sort of modification occurs on passage of classical scrapie into ARR/ARR sheep, such change does not result in the emergence of atypical scrapie or BSE and also reinforces the notion that the interaction between the infecting source and the *Prnp* genotype of the host has significant impact on the susceptibility to scrapie and on the disease phenotype [21].

## References

[1] Benestad SL, Sarradin P, Thu B, Schonheit J, Tranulis MA, et al (2003) Cases of scrapie with unusual features in Norway and designation of a new type, Nor98. Vet Rec 153: 202–208. 12956297 10.1136/vr.153.7.202

[2] Goldmann W (2008) PrP genetics in ruminant transmissible spongiform encephalopathies. Vet Res 39: 30–41. 18284908 10.1051/vetres:2008010

[3] González L, Dagleish MP, Bellworthy SJ, Siso S, Stack MJ, et al (2006) Post-mortem diagnosis of preclinical and clinical scrapie in sheep by the detection of disease associated PrP in their rectal mucosa. Vet Rec 158: 325–331. 16531580 10.1136/vr.158.10.325

[4] Jeffrey M, Martin S, Thomson JR, Dingwall WS, BegaraMcGorum I, et al (2001) Onset and distribution of tissue PrP accumulation in scrapie-affected Suffolk sheep as demonstrated by sequential necropsies and tonsillar biopsies. J Comp Pathol 125: 48–57. 11437516 10.1053/jcpa.2001.0476

[5] Jeffrey M, Begara-McGorum I, Clark S, Martin S, Clark J, et al (2002) Occurrence and distribution of infection-specific PrP in tissues of clinical scrapie cases and cull sheep from scrapie-affected farms in Shetland. J Comp Pathol 127: 264–273. 12443734 10.1053/jcpa.2002.0592

[6] McIntyre KM, Gubbins S, Goldmann W, Hunter N, Baylis M (2008) Epidemiological characteristics of classical scrapie outbreaks in 30 sheep flocks in the United Kingdom. PLoS One 3(12): e3994. 19098982 10.1371/journal.pone.0003994 PMC2601035

[7] Tongue SC, Pfeiffer DU, Warner R, Elliot H, del Río Vilas V (2006) Estimation of the relative risk of developing clinical scrapie: the role of prion protein (PrP) genotype and selection bias. Vet Rec 158: 43–50. 16415231 10.1136/vr.158.2.43

[8] Groschup MH, Lacroux C, Buschmann A, Luhken G, Mathey J, et al (2007) Classic scrapie in sheep with the ARR/ARR prion genotype in Germany and France. Emerg Infect Dis 13: 1201–1207. 17953092 10.3201/eid1308.070077 PMC2828083

[9] Ikeda T, Horiuchi M, Ishiguro N, Muramatsu Y, Kaiuwe GD, et al (1995) Amino acid polymorphisms of PrP with reference to onset of scrapie in Suffolk and Corriedale sheep in Japan. J Gen Virol 76: 2577–2581. 7595361 10.1099/0022-1317-76-10-2577

[10] Benestad SL, Arsac JN, Goldmann W, Noremark M (2008) Atypical/Nor98 scrapie: properties of the agent, genetics, and epidemiology. Vet Res 39: 19–26. 18187032 10.1051/vetres:2007056

[11] González L, Martin S, Houston FE, Hunter N, Reid HW, et al (2005) Phenotype of disease associated PrP accumulation in the brain of bovine spongiform encephalopathy experimentally infected sheep. J Gen Virol 86: 827–838. 15722546 10.1099/vir.0.80299-0

[12] González L, Chianini F, Martin S, Sisó S, Gibbard L, et al (2007) Comparative titration of experimental ovine BSE infectivity in sheep and mice. J Gen Virol 88: 714–717. 17251591 10.1099/vir.0.82426-0

[13] Andreoletti O, Morel N, LacrouxC, Rouillon V, Barc C, et al (2006) Bovine spongiform encephalopathy agent in spleen from an ARR/ARR orally exposed sheep. J Gen Virol 87: 1043–1046. 16528056 10.1099/vir.0.81318-0

[14] Bencsik A, Baron T (2007) Bovine spongiform encephalopathy agent in a prion protein (PrP) ARR/ARR genotype sheep after peripheral challenge: Complete immunohistochemical analysis of disease-associated PrP and transmission studies to ovine-transgenic mice. J Infect Dis 195: 989–996. 17330789 10.1086/512087

[15] González L, Dagleish MP, Martin S, Finlayson J, Sisó S, et al (2012) Factors influencing temporal variation of scrapie incidence within a closed Suffolk sheep flock. J Gen Virol 93: 203–211. 21918004 10.1099/vir.0.034652-0

[16] Ryder SJ, Dexter GE, Heasman L, Warner R, Moore SJ (2009) Accumulation and dissemination of prion protein in experimental sheep scrapie in the natural host. BMC Vet Res 5: 9–14. 19243608 10.1186/1746-6148-5-9 PMC2649917

[17] González L, Pitarch JL, Martin S, Thurston L, Moore J, et al. (In press) Identical pathogenesis and neuropathological phenotype of scrapie in valine, arginine, glutamine/valine, arginine, glutamine sheep infected experimentally by the oral and conjunctival routes. J Comp Pathol (In press: http://dx.doi.org/10.1016/j.jcpa.2013.06.006). 10.1016/j.jcpa.2013.06.006 24035191

[18] González L, Pitarch JL, Martin S, Thurston L, Simmons H, et al. (In press) Influence of polymorphisms in the prion protein gene on the pathogenesis and neiropathological phenotype of sheep scrapie after oral infection. J Comp Pathol (In press: http://dx.doi.org/10.1016/j.jcpa.2013.10.00). 10.1016/j.jcpa.2013.10.001 24342584

[19] Sisó S, Chianini F, Eaton S, Witz J, Hamilton S, et al (2012) Disease phenotype in sheep after infection with cloned murine scrapie strains. Prion 2: 1–10. 10.4161/pri.18990 PMC7082089 22421207

[20] Jeffrey M, González L, Espenes A, Press C, Martin S, et al (2006) Transportation of prion protein across the intestinal mucosa of scrapie-susceptible and scrapie-resistant sheep. J Pathol 209: 4–14. 16575799 10.1002/path.1962

[21] González L, Jeffrey M, Dagleish MP, Goldmann W, Sisó S, et al (2012) Susceptibility to scrapie and disease phenotype in sheep: cross-*PRNP* genotype experimental transmissions with natural sources. Vet Res 43: 55. 22748008 10.1186/1297-9716-43-55 PMC3460791

[22] Eaton SL, Rocchi M, González L, Hamilton S, Finlayson J, et al (2007) Immunological differences between susceptible and resistant sheep during the preclinical phase of scrapie infection. J Gen Virol 88: 1384–1391. 17374786 10.1099/vir.0.82197-0

[23] Chianini F, Sisó S, Ricci E, Eaton SL, Finlayson J, et al (2013) Pathogenesis of scrapie in ARQ/ARQ sheep after subcutaneous infection: Effect of lymphadenectomy and immune cell subset changes in relation to prion protein Vet immunol immunopath. 152: 348–358. 10.1016/j.vetimm.2013.01.005 23398720

[24] Jeffrey M, González L, Chong A, Foster J, Goldmann W, et al (2006) Ovine infection with the agents of scrapie (CH1641 isolate) and bovine spongiform encephalopathy: Immunochemical similarities can be resolved by immunohistochemistry. *J Comp Pathol* 134: 17–29. 16324707 10.1016/j.jcpa.2005.06.005

[25] Spraker TR, ORourke KI, Balachandran A, Zink RR, Cummings BA, et al (2002) Validation of monoclonal antibody F99/97.6.1 for immunohistochemical staining ofbrain and tonsil in mule deer (*Odocoileus hemionus*) with chronic wasting disease. J Vet Diag Invest 14: 3–7. 10.1177/104063870201400102 12680636

[26] Choi JK, Park SJ, Jun YC, Oh JM, Jeong BH, et al (2006) Generation of monoclonal antibody recognized by the GXXXG motif (glycine zipper) of prion protein. Hybrid (Larchmt.) 25: 271–277. 10.1089/hyb.2006.25.271 17044782

[27] Brun A, Castilla J, Ramirez MA, Prager K, Parra B, et al (2004) Proteinase K enhanced immunoreactivity of the prion protein-specific monoclonal antibody 2A11. Neursosci Res 48: 75–83. 10.1016/j.neures.2003.09.004 14687883

[28] Jacobs JG, Bossers A, Rezaei H, van Keulen LJ, McCutcheon S, et al (2011) Proteinase K-resistant material in ARR/VRQ sheep brain affected with classical scrapie is composed mainly of VRQ prion protein. J Virol 85: 12537–12546. 21917981 10.1128/JVI.00448-11 PMC3209378

[29] Thuring CMA, van Keulen LJM, Langeveld JPM, Vromans MEW, van Zijderveld FG, et al (2005) Immunohistochemical distinction between preclinical bovine spongiform encephalopathy and scrap[ie infection in sheep. J Comp Pathol 132: 59–69. 15629480 10.1016/j.jcpa.2004.06.004

[30] Jeffrey M, González L (2004) Pathology and pathogenesis of bovine spongiform encephalopathy and scrapie. In Mad cow disease and related spongiform encephalopathies Ed. DA Harris, Pub Springer –Verlag Berlin, Heidelberg. pp 65–98.

[31] Elsen JM, Amigues Y, Schelcher F, Ducrocq V, Andreoletti O, et al (1999) Genetic susceptibility and transmission factors in scrapie: detailed analysis of an epidemic in a closed flock of Romanov. Arch Virol 144: 431–445. 10226611 10.1007/s007050050516

[32] Stack M, González L, Jeffrey M, Martin S, Macaldowie C, et al. (2009) Three serial passages of bovine spongiform encephalopathy in sheep do not significantly affect discriminatory test results. J Gen Virol, 90: , 764–768. 10.1099/vir.0.005983-0 19218224

